# Rasmussen’s Aneurysm in Active Pulmonary Tuberculosis: A Case Report

**DOI:** 10.7759/cureus.68148

**Published:** 2024-08-29

**Authors:** Khalid Y Fadul, Ahmed Alsayed, ELMustafa Abdalla, Rawan S Mohamed, Amjad M Salman, Ahmad Meer, Abdalla Fadul

**Affiliations:** 1 Emergency Medicine, Hamad Medical Corporation, Doha, QAT; 2 Internal Medicine, Hamad Medical Corporation, Doha, QAT; 3 Medicine, Hamad Medical Corporation, Doha, QAT; 4 Neurology, Hamad Medical Corporation, Doha, QAT

**Keywords:** pulmonary artery pseudo aneurysm, chest computed tomography, pulmonary tuberculosis sequelae, massive hemoptysis, rasmussen’s aneurysm

## Abstract

Hemoptysis is a common presenting symptom of pulmonary tuberculosis (TB). Rasmussen aneurysm can present with severe hemoptysis, which is usually diagnosed using computed tomography (CT) angiography. A false aneurysmal dilatation of the pulmonary artery is known as a Rasmussen aneurysm. It occurs due to a gradual weakening of the arterial wall adjacent to pulmonary cavitation. Computed tomography angiography of the chest is the standard diagnostic technique for Rasmussen aneurysm. An early angiographic or surgical procedure with vascular embolization is recommended following a definitive diagnosis. We present a 29-year-old woman whom the medical commission referred due to a cavitary lesion on a screening chest X-ray. Hospital admission was preferred for the *Mycobacterium tuberculosis* infection workup, which revealed radiological evidence of the Rasmussen aneurysm. The patient was eventually treated as a case of active tuberculosis on a radiological basis via the decision of the local infectious disease (ID) team. The most common symptoms reported in patients with tuberculosis infection are hemoptysis, cough, low-grade fever, night sweats, and weight loss. Hemoptysis can rarely originate from the Rasmussen aneurysm of the pulmonary artery. However, hemoptysis is the predominant symptom in chronic cavitary tuberculosis with Rasmussen aneurysm. A CT pulmonary angiogram (CTPA) is considered the imaging modality of choice to confirm the diagnosis of Rasmussen aneurysm. Fatal hemoptysis is one of the consequences of a Rasmussen aneurysm if it is massive and not treated promptly. Confirming the diagnosis with proper follow-up is essential to preventing devastating outcomes.

## Introduction

In the industrialized world, the incidence of pulmonary tuberculosis (TB) is much lower than in underdeveloped countries [1]. Risk factors include diabetes, aging, long-term corticosteroid use, tumor necrosis factor-alpha (TNF-α) blockers, vitamin D receptor polymorphisms, and polymorphisms in the IL-12 and IFN-γ genes [2]. Hemoptysis is a common presenting symptom of pulmonary tuberculosis; however, massive hemoptysis only happens in approximately 8% of cases, with a 5-25% death rate linked to it [3]. Rasmussen aneurysm, a false aneurysmal dilation of the pulmonary artery, can manifest as severe hemoptysis [4]. A progressive weakening of the arterial wall next to pulmonary cavitation is the usual mechanism of Rasmussen aneurysm formation [5]. In 1939, according to Auerbach's research, 45 of the 114 corpses with chronic cavitary tuberculosis had Rasmussen's aneurysms [6,7]. The recommended diagnostic method for pulmonary artery pseudoaneurysm is computed tomography (CT) angiography [8]. After a precise diagnosis, early angiographic or surgical procedures with endovascular embolization are advised [9]. Here, we describe a case of Rasmussen's aneurysm seen on CT thorax in a patient who had previously contracted tuberculosis, which resulted in lung cavity scarring and fibrosis.

## Case presentation

A 29-year-old Indonesian lady with a past medical history of migraine and successfully treated pulmonary TB ten years ago was referred to the medical commission upon arrival from Indonesia due to an abnormal chest X-ray result. The patient was completely asymptomatic. She denied fever, cough, sputum production, night sweats, weight loss, and shortness of breath. She has no recent sick contacts.

**Figure 1:**
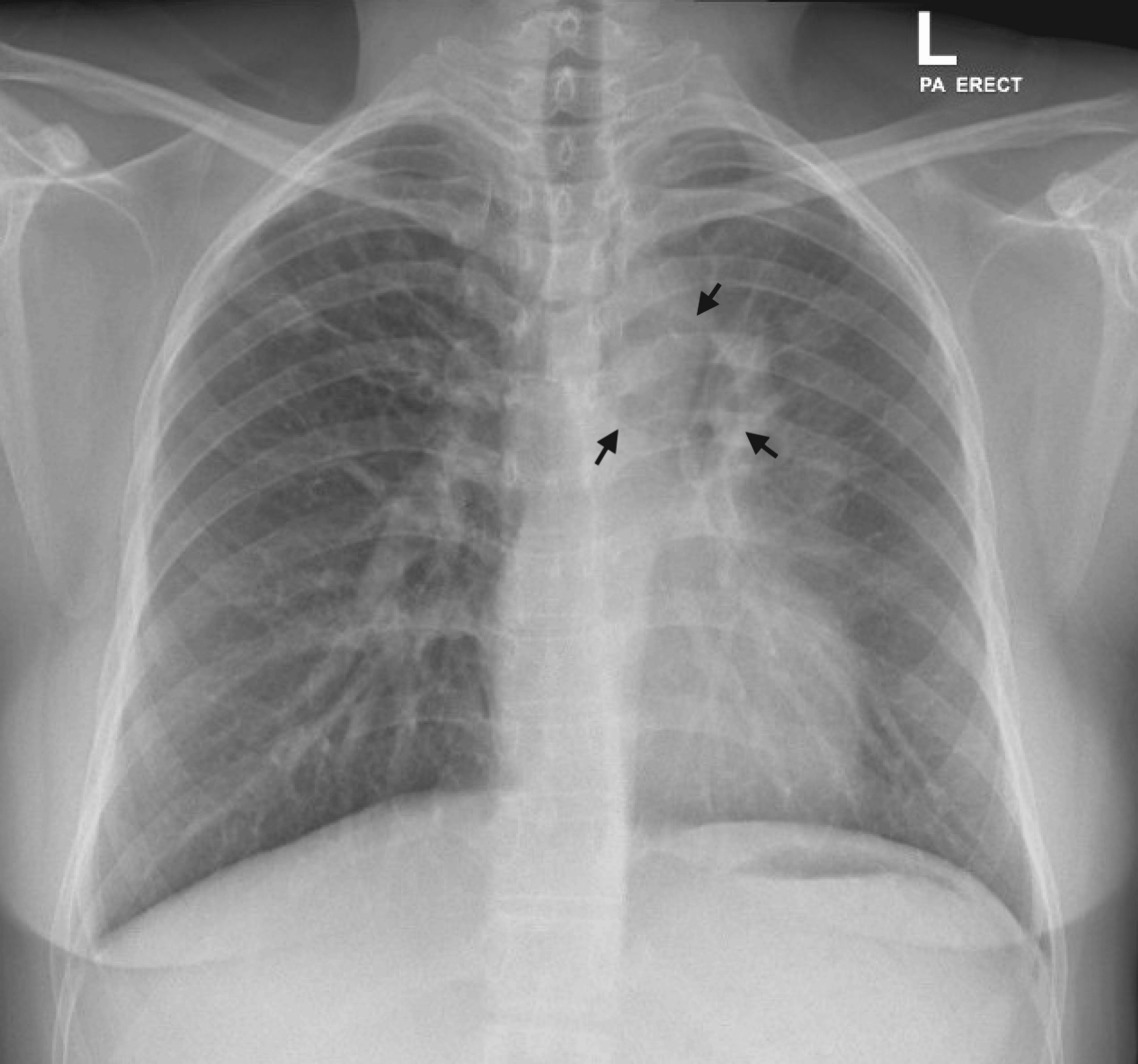
A chest X-ray showing a cavitary lesion in the left upper lung zone with hazy opacity seen in the left hilar region.

Upon assessment, she was vitally stable, and a physical examination showed decreased breath sounds and vocal resonance over the left upper-mid zone. Initial basic labs were normal, apart from a low vitamin D level (17 ng/ml). A chest X-ray revealed a cavitary lesion in the left upper lung zone, with hazy opacity seen in the left hilar region (Figure 1). 

**Figure 2:**
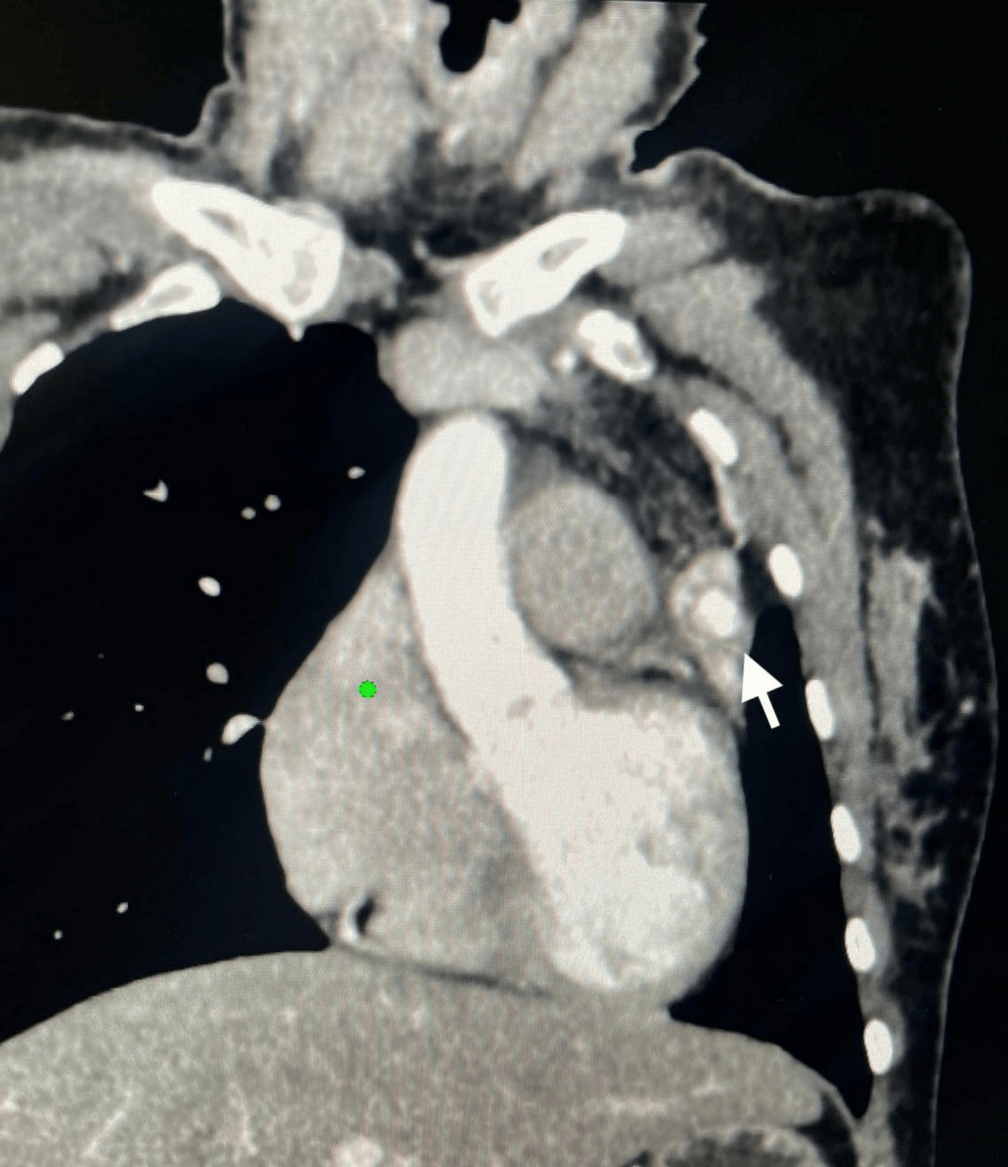
A coronal view of a chest CT scan revealing a hyperdense rounded lesion in the collapsed left upper lung lobe.

**Figure 3:**
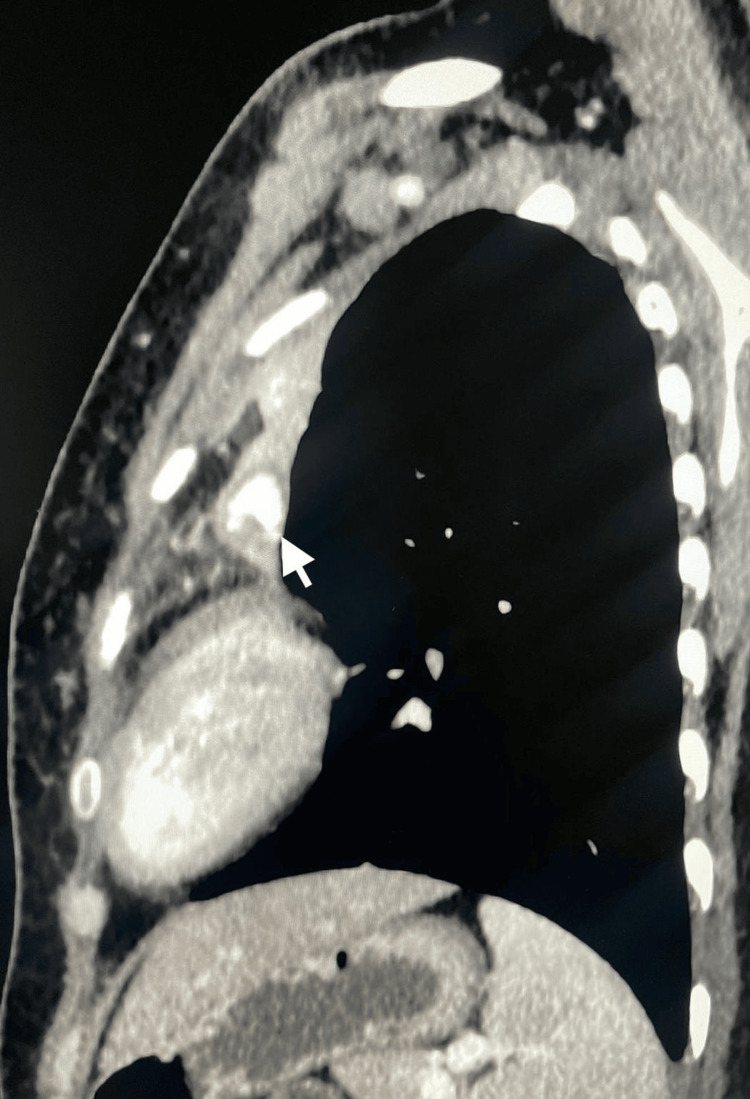
A sagittal view of a chest CT scan revealing a hyperdense rounded lesion in the collapsed left upper lung lobe.

**Figure 4:**
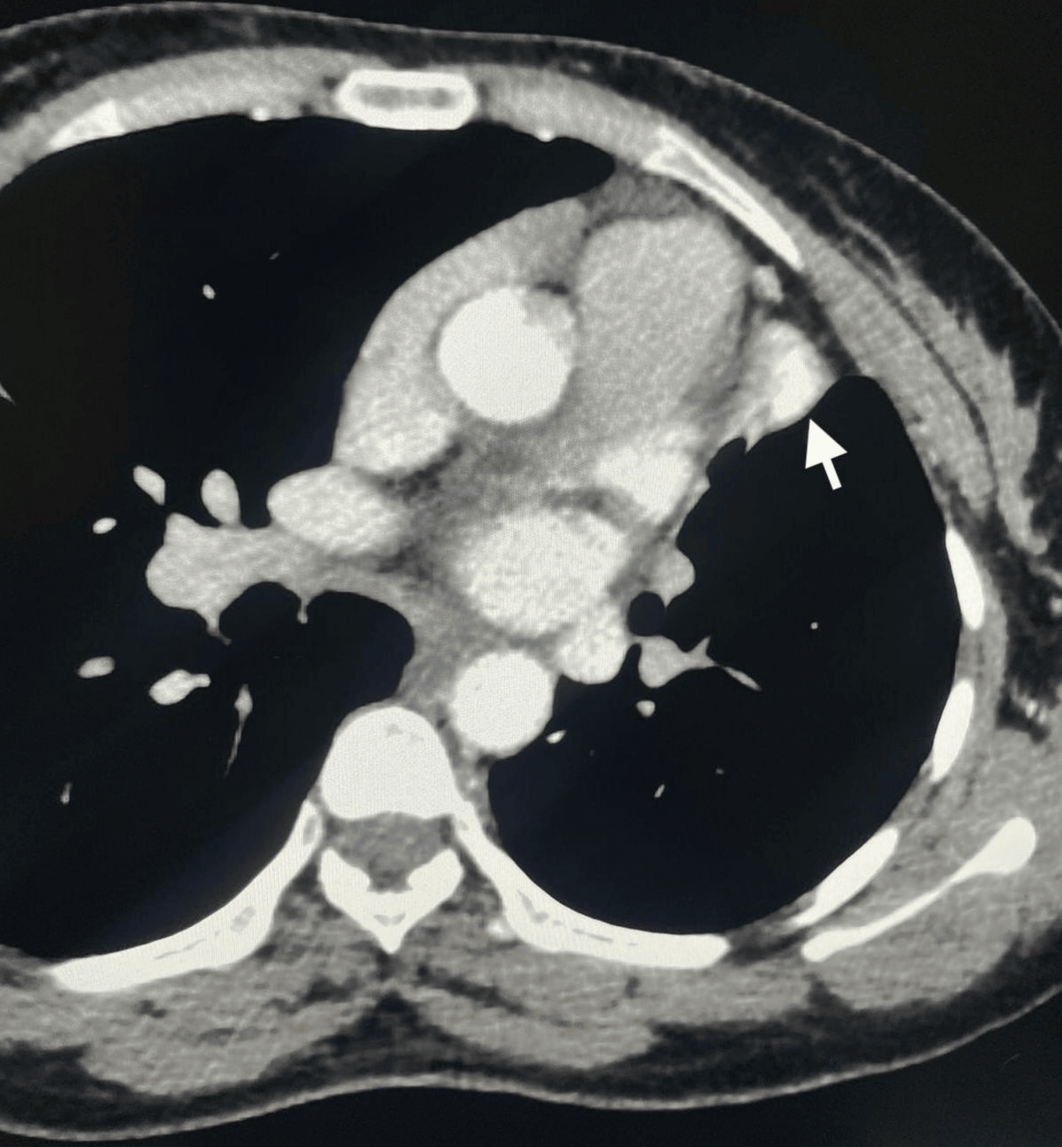
An axial view of a chest CT scan revealing a hyperdense rounded lesion in the collapsed left upper lung lobe.

Two sets of acid-fast bacilli (AFB) smears from the sputum were negative, sputum TB PCR was negative, and sputum TB culture came negative at follow-up. A Quantiferon TB Gold Plus test was positive. CT imaging of the thorax with contrast was performed on day 8 of admission to obtain further details regarding lung parenchyma. CT thorax showed bilateral upper lung lobe fibrosis and scarring with complete left upper lobe volume loss, most likely sequelae of an old granulomatous infection. The CT thorax also showed a hyperdense, rounded lesion in the collapsed left upper lung lobe, raising the possibility of a Rasmussen aneurysm (Figures 2-4).

The infectious disease (ID) team was consulted on day 9 of admission for suspected active pulmonary TB infection; they recommended bronchoalveolar lavage (BAL). BAL was performed on day 11, and the BAL TB PCR and AFB smear were negative later. BAL was also negative for malignancy and granuloma.

The ID team eventually decided to treat the patient as having active pulmonary TB based on the radiological findings (before the final BAL result); hence, TB treatment was initiated on day 12 of admission. The patient was discharged home on anti-TB medications. She was referred to the TB clinic for follow-up. Confirmation CT pulmonary angiography (CTPA) for Rasmussen aneurysm was planned to be performed as an outpatient.

## Discussion

Pulmonary TB is a bacterial infection caused by *Mycobacterium tuberculosis* that spreads through air droplet inhalation, typically with pulmonary and extrapulmonary manifestations [10]. TB incidence in the year 2020 was around 127 cases per 100,000 population; most of these cases were in the WHO region of Southeast Asia (43%), Africa (25%), and the Western Pacific (18%) [11]. The most common symptoms reported in patients presenting with TB are hemoptysis, cough, low-grade fever, night sweats, and weight loss [10,12,13]. Its mortality rate in 2020 was estimated to be around 17/100,000 and 2.7/100,000 in HIV-negative and HIV-positive populations [11]. Our patient's main presentation was hemoptysis and an abnormal CXR. She had no systemic symptoms such as fever, weight loss, or night sweats.

Rasmussen aneurysms, first described in 1868 by Rasmussen and Moore [14,15], are inflammatory pseudo-aneurysms that affect a branch of the pulmonary artery close to the tuberculous cavity [1,10,16]. It is caused by progressive weakening of the arterial wall as tunica adventitia and tunica media are replaced by granulation tissue and eventually fibrin [10,12,16]. It usually affects the upper lobes [17]. RA is reported to have a 5% prevalence among patients with chronic cavitary TB [5,9,12]. Hemoptysis is the predominant symptom with a high risk of rupture because of the inflammatory processes in the arterial wall [18]. Hemoptysis from RA can be massive, leading to potential life loss if not detected and treated promptly [1,9,10,12,16,18,19]. On chest radiographs, it is manifested as an expanding mass, lung nodule, intracavitary protrusion, or hilar enlargement [16,20]. Chest radiographs, CT thorax with contrast, and bronchoscopy can be done [13], but CT pulmonary angiography is considered the imaging modality of choice [9,13,17] as it helps to confirm the presence of RA and identify its size, characteristics, and location; MRI is a feasible alternative in cases where CTPA is contraindicated (e.g., renal insufficiency and iodinated contrast allergy) [17]. CTPA was not performed in our case as it was planned as an outpatient, and the patient did not follow up as planned.

Mild to moderate hemoptysis secondary to RA can be controlled by anti-TB medications [13]; however, in the event of leaking RA, the priority in management is to secure the airway and ensure appropriate oxygenation [15], followed by control of bleeding using trans-arterial embolization; if the embolization is unsuccessful, surgical intervention might be warranted [13]. One of the limitations was the unavailability of the patient's medical records from her previous TB infection. This was due to her residence in her home country at the time, which made details about her previous TB infection and treatment unavailable. Additionally, the history she provided was not completely reliable due to the language barrier factor. Another limitation was the absence of a CTPA report, as she was discharged with plans for it to be done as an outpatient.

## Conclusions

Hemoptysis is common among tuberculosis patients. It can result from bronchiectasis originating from bronchial artery or cavitary lesions. Rasmussen aneurysm is one of the rare causes of hemoptysis in chronic cavitary tuberculosis patients. Hemoptysis due to Rasmussen aneurysm can range from mild to severe and fatal. Rasmussen aneurysm can be discovered incidentally in tuberculosis patients who are not complaining of hemoptysis during the radiological workup of tuberculosis. Diagnosis confirmation and proper follow-up are essential to prevent the devastating outcome, especially in patients whose presentation did not necessitate an urgent therapeutic intervention. We are hoping to improve physicians' awareness of this rare cause of hemoptysis in tuberculosis patients by reporting this case.
